# Practical Remarks on Amputations, Fractures, and Strictures of the Urethra

**Published:** 1830-04-01

**Authors:** 


### XIII.

**Practical Remarks on Amputations, Fractures, and Strictures of the Urethra.** By *Stephen Love Hammick*, Surgeon Extraordinary to the King, and late First Surgeon of the Royal Naval Hospital at Plymouth.

We were a good deal disappointed on turning from the title page to the preface of this work, to find that these practical remarks were no more than clinical observations delivered to the surgeons' mates of the navy at various periods of the late war. A surgeon who had had such a theatre of experience, and who attained such vast provincial renown as Mr. Hammick acquired, might have produced something of a higher character than the present volume of elementary details, delivered to the juniors of the naval medical profession. Surely a man of half the talents which the author is supposed to possess, might, at his time of life, have addressed the very heads of the surgical profession, on topics of the highest interest. Were we inclined to indulge in satire, on this occasion (but our readers are aware that we never deal in that article) we should say that, instead of a spank new frigate, just off the stocks of Devonport, we found, on boarding the vessel in question, an old friend with a new face—a cruiser fitted out in hamoaze with a cargo of old naval stores for the London market. There is not a plank in her decks that is not as familiar to our eye as the romantic promontories round Catwater and Causand Bay—not a cask in her hold in which we do not recognize an old *mess-mate*, SALT-JUNK—not a biscuit in her bread-room untenanted by our quondam companions in arms—the woevils.

> Et quodcunque semel chartis illeverit, omnes
> Gestiet a furno redeuntes scire lacuque,
> Et pueros et anus.

But to drop metaphor. It must be very evident to our readers that Mr. Hammick's work is not adapted for analysis in this Journal. Its elementary character forbids such a mode of procedure, whilst, on the other hand, it must be supposed, that mixed up with much common-place matter, are many practical and valuable remarks. We shall select some of them as well as we can, but our readers may believe that picking out insulated passages can be pleasant neither to ourselves, nor the author. The first part of the work is dedicated to the subject of amputations, the second to that of fractures, the third to the consideration of strictures of the urethra. We are sorry to say that there is neither table of contents, nor index, nor any other mode of arrangement that we can discover; indeed Mr. Hammick would seem to have taken a hint in book-making from the Sybill of old, and selecting some windy day in Plymouth Sound, tossed the notes of his Clinical Lectures into the air, from whence, by good luck, they fell into their present form.

### AMPUTATIONS.

Our author never performed amputation at the hip-joint, but assisted at that performed by Mr. Brownrigg in the Military Hospital of Plymouth, and observes, that it is an operation which no competent surgeon would shrink from attempting, when circumstances render it necessary. The plan which he would pursue if called upon to operate differs from that adopted by Mr. Brownrigg, and is as follows :—

"In the removal of the thigh at the hip-joint, you only have the power of compressing the femoral artery at the groin ; consequently, if all the muscular parts on both sides are divided before the bone is taken from the socket, which was the case in this operation, you must cut through the obturator artery, as well as those branches which come out of the pelvis at the posterior part, consequently you have several vessels bleeding at the same moment, without the power of compression ; and from the thigh not being removed from the acetabulum, you have only the slight space formed by the gaping of the cut muscles all around the bone to wedge in your hand, and get hold of the mouths of the bleeding vessels,—which mode of operating places you between two difficulties, either of allowing several of these arteries to bleed, whilst you are employed in cutting open the capsular ligament, dividing the round one, and removing the thigh from the joint, or of attempting, through this narrow muscular space, to secure the vessels; and should there be any loss of time from their retraction within the muscles, or from any of the impediments which every now and then occur in the taking up of arteries, a considerable quantity of blood may be lost, which, if not immediately fatal, may so far debilitate the patient as to render him incapable, should the stump not heal kindly, of struggling through long periods of suppuration. Now, the method I should recommend would be this:—I should place my patient on a table, with his body half-raised, leaning back against the breast of a person sitting behind him; the patient's lower limb should hang over the edge, so that the groin should be fairly exposed, and on the stretch; the thighs are to be separated, so that I might, if I chose, be able to sit down on a stool in front, with my foot resting on the bar of the table, of such a height as to allow the right elbow to rest on the knee. I should direct a steady assistant to place three fingers of his right hand close to the femoral artery, on its iliac side, as it emerges from under Poupart's ligament, pressing them down, so as in some degree to protect the vessel, and, if necessary, to be in readiness to make pressure on the artery: I should then make an incision merely through the integuments with a scalpel of the largest size, from the iliac edge of my assistant's fingers, carrying it downwards and outwards to below the trochanter major, so as to form the line of the outer flap, which is to terminate near the tuberosity of the ischium: this being done, the assistant should shift his fingers to the pubic side of the femoral artery, with the same view of protection as he did before on the iliac side; then I should mark out the intended flap on that side by an incision merely through the integuments: these lines would very nearly join in front, excepting that part over the femoral artery covered by the assistant's fingers, but they are to meet behind, carrying them higher or lower, according to the muscularity of the subject. If I was then satisfied that this track through the integuments offered a fair chance of forming a good stump, I should follow it; if not, I should alter my line, by carrying it a little further up or down. This being settled, the assistant's pressure is again to be on the iliac side of the artery, when, by a bold stroke, I should go down by the line already made in the integuments to the bone on the outer side, and immediately dissect up, and turn back the muscles, to form the outer flap, and should secure in my progress any arteries that might be troublesome. The next steps would be to cut through the capsular ligament, so as to lay open the joint,—then to divide the round ligament, and the limb would immediately drop a little; when, with a smaller scalpel, carried as close to the bone as possible, the attachment of all the muscles should be divided; and as soon as I could lay hold of the head of the femur with my left hand, I should draw it outwards from the joint, so as to allow of passing a small amputating knife close to the bone, on its inner side, to be carried down till I came to the line of my inner incision, and then resigning the head of the femur to an assistant, I should place the fore-finger of my left hand on the point of my knife, and dash it directly out in the groove previously made in the integuments, so that the femoral artery would not be cut through till the moment of the separation of the limb from the body; then instantly seize hold of the artery, directing the assistant to keep strong pressure on it as it lays over the edge of the pubis: at this time the other assistants might be pressing on, or securing the mouths of the other vessels, which would not be long in tying after the femoral artery was in safety; so that, in fact, by this method, none of the more important arteries would be cut through till the division of the muscles on the inner side, which is to separate the thigh; and then we should have an open surface, and nothing to prevent our meeting the bleeding vessels fairly: these being all tied, the lips of the wound are to be brought together, either transversely or longitudinally, whichever at the moment appears the easiest and most convenient direction; and let them be kept in contact by adhesive straps, with central holes, to allow of the ligatures to hang out, and any matter to escape. Spermaceti ointment, spread on lint, is to be applied, and over it a square piece of calico, which is to be fastened by tapes to a circular roller on the loins. In Mr. Brownrig's case the lips of the stump were brought together transversely, and they formed a most perfect line. *An assistant should be constantly by the bed-side of the patient till the ligatures are away;* the period and method of dressings are very nearly the same as those for an amputated thigh." 21.

On the foregoing proposal we have nothing further to remark, than that we would rather be the operator than the assistant; for to be constantly at the bed-side of a patient till all the ligatures come away, would be no joke. Mr. Hammick deprecates scraping the cartilaginous lining of the acetabulum with a scalpel, and disapproves of the plan recommended by Mr. Charles Bell, of commencing the operation by cutting down on and securing the femoral artery.

With respect to amputation of the lower part of the limb, our author would run many risks for the advantage of operating below the knee, that joint being so valuable a point d'appui for the wooden leg. It occurred to him several years ago, that, after having sawn through the bone, it was discovered to be so diseased in its inferior part, that although another portion of two inches was removed higher up, he was obliged instantly to amputate above the knee-joint; the patient fortunately did well. Since that period Mr. Hammick has cut off some legs where the risk of the same accident was equally great, and yet it has not happened. After describing the steps of the operation, he makes the following judicious observations:—

"Our principle, therefore, in amputating a leg is the same as for the thigh, —first, to separate the integuments by a circular incision, and then to draw them up from the muscles, just touching, if necessary, their cellular connexion with the point of the kife, so as to detach them, more or less, according to the muscularity or emaciation of the limb. It is more necessary to have a greater freedom of integuments to cover the face of a leg than a thigh stump. In order to avoid the sharp edges of the tibia, we prefer sawing the bone separately, so that the fibula may be somewhat shorter than the tibia, the benefit of which you will discover in dressing up the stump, particularly if the limb be greatly emaciated; when, by the common method of sawing both bones at once and making them of the same length, you occasionally find the point of the fibula projecting against the inner side of the integuments, so that it brings on pain and inflammation, and sometimes the bone will ulcerate through, unless you take the greatest care, when you bring forward the integuments to the central longitudinal line of your stump, not to make pressure on it. If you intend to saw the bones separately, it is better to get through the fibula first, which avoids the risk of splintering that small bone, which might happen by the weight of the limb on turning it to get at the fibula, supposing the tibia had been sawn through. For comfort and convenience, a stump below the knee cannot be too short; taking care to keep clear of the joint, and that it may be so formed that the tibia may be cut through about an inch and a half to two inches below the tubercle, which will keep you free of the tendinous insertion of muscles, which, if divided, might slough. Sailors have frequently requested me to make their stumps as short as possible, so that when the knee-joint rested on the wooden leg, there might be but little projection from behind; for, when the stump is long, its weight and swinging about retard and fatigue in walking:

two men, who had lost their legs in the East Indies, came to me in this hospital at one time, whose stumps were so long, having been cut off just below the calf, that they were rendered almost helpless, and at their earnest entreaty I removed them to the usual shortness, for which they were very grateful. Take care, however, not to go too close up to the knee; I had once a patient sent to me eleven days after his leg had been amputated on board in the heat of battle, and so close was it done to the head of the tibia, that the joint was freely exposed, and it became necessary to cut off the limb above the knee, so save his life. We are of opinion that it is more to the comfort, besides incurring less risk, for all persons to have the limb amputated at the usual place below the knee than to perform it, even in such cases as would admit of it, any where below the calf; people of affluence, who can afford an expensive and complicated artificial limb, instead of the common wooden leg, may be disposed to have the operation lower down, but for others not similarly situated, there can be no doubt of the spot that is best to be selected, and the risk is certainly less, with a better prospect of a sound stump." 30.

Speaking of amputation at the shoulder-joint, Mr. H. observes that the frequency with which some surgeons have performed it, in comparison with others, who have had the care of many more surgical patients, must induce us to suspect that it has often been resorted to, when the limb might have been amputated below. This, of course, is bad practice, for, not to mention the greater danger, the body is more awry after the shoulder-joint operation than that below the articulation; and besides, if the stump in the former case turn out amiss, no resource remains in amputation higher up. During the operation, our author is of opinion that the patient should be laid on a table, with the arm supported over the edge. When seated on a chair, he winces under the pressure of the key on the subclavian artery, and sometimes gradually slides down till he is nearly on the floor.

In amputation of the fore-arm, the tourniquet is to be placed on the arm as near the elbow as possible, and the longer the stump of the fore-arm the better. The patient sits on a form, leaning back against the chest of an assistant, who holds the elbow half-flexed very securely, and grasps the integuments of the fore-arm, whilst another holds the lower parts by the hand or the wrist, as is most convenient; the fore-arm is maintained half-supine, with the integuments on the stretch. If the operation is to be near the carpus, the first incision should go at once down to the bones, the assistant forcibly retracting the parts; and when these are free from the radius and ulna, and drawn upwards an inch and a half, the catlin is to be passed between the bones "at the upper part of the retraction." If the knife were inserted lower down, the muscles would require to be slit up from between the bones, by which they would be too much divided, and the inter-osseous artery would be in danger of being cut too high. The retractor is next to be inserted, the fore-arm pronated, and the bones sawn through, the vessels tied, and the stump to be dressed, with the fore-arm half supine. The patient is to be put to bed with the shoulder supported, the elbow bent, and the fore-arm prone, resting on pillows; after the fifth day, the patient may get to the sofa; and, in a day or two more, may walk about with his arm in a sling, supported on a pillow.

On concluding his directions with respect to the particular amputations, Mr. Hammick informs us that, during his service, he has performed two hundred and eighty-seven amputations, of which sixteen have been fatal. We are sure that this is not meant as a gasconade, but ill-natured persons might impute to Mr. Hammick a disposition to the puff oblique, of which he is utterly innocent. Within the last eighteen years, the number of amputations has diminished very considerably, in consequence of the malignant ulcer having ceased its destructive ravages in the navy.

Our author disapproves of ligatures of single silk, and thinks that the practice has been pushed by some surgeons a little too far. In his opposition, he would seem to be rather prompted by his fears than by actual experience of their danger, for he confesses that he never saw hæmorrhage from their use, but, in two instances, was very much afraid of it. We must confess that we have not been witnesses, any more than Mr. Hammick, of disastrous consequences from single silk ligatures, nor do we participate altogether in his repugnance to their use. Mr. Hammick believes, and the creed, we fancy, is somewhat heterodox, that larger ligatures are more easily and earlier thrown off, in proof of which position he cites the case of "a youngster," in whom three single silk ligatures remained in the stump after amputation of the thigh for ten months, and at length appeared to rot away. In three instances, where the ends of the ligature were cut off close to the knot, and the edges of the wound attempted to be closed over it, troublesome abscesses succeeded. The experience of the best surgeons is in accordance with our author's on this point.

"Having made these observations to you on the various ligatures, the following is our method:—we use either silk or fine thread, generally taking the latter, making our ligatures from one to eight threads in thickness, waxing them a little into a round form, just to connect them slightly together, so that they may be tied without the risk of being of unequal lengths, or twisting up under the knot, it being absurd to wax them to any great degree to prevent their rotting, for they may remain months without being in the least injured, and where much wax is used you will see it crack or scale off at the time of tying your ligature." 47.

It signifies nothing that this kind of ligature is not commonly employed, for it by no means follows that the established practice is the best; we therefore recommend surgeons to make trial of Mr. Hammick's method, for his experience in these matters renders his opinions worthy of attention. As soon as the limb is removed, whilst the tourniquet is kept tightly screwed, spunge the surface of the stump well with warm water, and secure the arteries in succession, taking care to look that the side of the vessel has not been injured by the knife or catlin, above where you place the ligature. The following observations on the mode of unscrewing the tourniquet are, as far as we know, original and ingenious.

"Having satisfactorily secured all the important arteries of the stump, boldly unscrew the tourniquet, so that the blood may come down with a sort of rush, and you will immediately afterwards see the ligatures in movement by the pulsation of the arteries; then secure any of the smaller ones that may require it. My object in rapidly unscrewing the tourniquet after the large arteries are secured, is, that the rush of blood may force any of the secondary ones which may have retracted during the operation into the muscles, to bleed; otherwise they may lie concealed and undetected until the patient has been two or three hours in bed; when, re-action taking place, from the fear of the operation having subsided, and the rallying of the system from the loss of blood and faintness, hæmorrhage comes on, and you are frequently obliged to open the stump to secure the vessel: if not, the blood thrown out forms a large cake, acting as an extraneous body within its lips, preventing union, not only by the first intention, but frequently doing much more mischief; particularly if it be in a stump where there has been preserved too much integument and too little muscle. Now, what is the effect of a cautious and gradual slackening of the tourniquet, which you see so commonly done? Why, that the face of your stump becomes a bleeding surface, so that innumerable vessels are tied. How common is it for the surgeon, after having secured all the vessels he could get hold of, whilst the tourniquet was tight, to hear him say to his assistant, 'Pray unscrew very gradually; but mind you stop the very moment I tell you.' Well, after being slackened a turn or two, an oozing takes place all over the stump, and the surgeon immediately cries out for the tourniquet to be screwed tight, as the patient is bleeding fast. Now, mark what he has done: he has just slackened the tourniquet enough to allow the blood to pass down by the important arteries, and has kept it quite tight enough to prevent the blood getting back by the veins, or the anastomosing branches, so that the whole is rendered a bleeding surface; and a timid surgeon will go on securing vessel after vessel, to his own astonishment, the greater part of which need not have been touched; and the hæmorrhage would have ceased on boldly taking off the tourniquet, and spunging the face of the stump with cold water. I have found that the slower the incisions have been made in removing the limb, and the more emaciated the patient, the more numerous, in general, will be the bleeding vessels. In amputations, as well in large incised wounds, by accurately examining the surface, you will detect arteries of the second and third order occasionally protruding beyond the muscular parts at least the length of an eighth or a quarter of an inch, pulsating violently, though without any flow of blood. Their mouths, on close inspection, appear shut, with their ends forming an obtuse point; and this happens more frequently in very muscular subjects, where the operations have been very dexterously performed. Now, should you pass over these arteries without securing them, you will find, that within six hours of the patient having been returned to bed, by the muscular parts relaxing from the return of warmth, hæmorrhage will pour forth from these vessels. You had better, in all such cases, snip off the end of the projection, and immediately a start of blood will take place, convincing you of the necessity of tying the artery before you have closed your stump." 51.

If the vessel should be found in an ossified or diseased state, it should be secured by passing a needle, in doing which it is desirable to include a pretty good cushion of surrounding substance, to prevent its being cut through or yielding, which Mr. H. has "seen it do like a rotten pear." Sometimes when even the surrounding substance has been so sloughy and soft as to give way, the introduction of a piece of lint within the noose of the ligature has been perfectly successful. When the needle is used, we should be particularly careful not to wound the vessel with its point, for obvious reasons. Mr. Hammick particularly cautions surgeons not to grow fidgetty and uneasy if a ligature on an artery is retained beyond the usual time, and warns them never to employ force in its removal. Twice from such a cause has he seen the patient's life in imminent danger. In the first case, the stump was dressed by the surgeon of a gun-brig on the third day after amputation, and every ligature forcibly removed, with the effect of producing the greatest pain; extensive inflammation and sloughing followed; tetanus was threatened; and it was several months before the stump was healed. In the second case, similar violence in attempting to remove the last ligature from the stump of an upper arm, was likewise followed by extensive inflammation and sloughing. In cases of unusually long retention of the ligature then, the practice is to keep the stump clean and tranquil, and to wait with patience till nature choose to permit its separation. If, however, the patient insist on some force being applied, put the ligature only gently on the stretch around a piece of flat bougie, which is to be secured beyond the edge of the stump by a slip of adhesive plaster, repeating it at every dressing. In the case of "a nice little lad" from the Impregnable, whose thigh was removed for the malignant ulcer, our author gently touched the ligature on the femoral artery with the forceps on the tenth day, when he shrieked out with pain, violent tetanic spasms instantly succeeded, and in forty-six hours he was dead. On dissection no nervous twig was found included in the noose with the artery, nor was any thing discovered to account for the fatal result.

### FRACTURES.

Having concluded the subject of amputations, we now pass to that of Fractures. The splendid work of Sir Astley Cooper on this part of surgery will, like the rod of Aaron, swallow up for many years to come the interest that might attach to the productions of authors of less calibre, and we shall therefore merely glance at Mr. Hammick's observations on the non-union of fractured bones. Mr. H. believes that the want of union is generally attributed to too few causes, mere motion of the fractured ends not being by any means the whole and sole one.

"We, and most surgeons of the present day, use splints of such a length as firmly to secure the heads of the bones, so that, by their being fixed, any motion is precluded to the fractured parts. There are some, however, who apply splints so short, that they extend only a little distance each way beyond the fracture; and let these be bound ever so tight, they cannot preclude a freedom of motion to the broken bone; and this, we shall find, is a frequent cause of a retarding, if not of a total want of union. The older surgeons were often afraid of being disgraced by such a superabundance of callus as might cause any outward deformity; and to prevent this from occurring, they bound down short splints very tightly over the fracture: we now never employ them with that view, from knowing that such a deposit of callus very much strengthens the limb, and as the exuberance is concealed, it can be of very little moment; besides, the binding down of splints in this way does not prevent the formation of the callus, though it might give it another and a deeper direction; and although it might hinder it from shewing itself so prominently outwards, yet the practice did a great deal of harm; for when nature would have deposited it in the most commodious manner for the strength and security of the limb, it was forced by this interference into the interstices of the muscles, whereby their action was ever afterwards considerably impeded." 114.

Disturbance of the limb will often prevent union, a good illustration of which is given by our author in the case of a gallant general officer, who got his aide-de-camp to pull long and lustily at his foot every night and morning, after fracture of his thigh, in order to prevent any shortening of the limb! Another cause of non-union is one the very *antipous* of too much motion, viz. too little. Some years ago, Mr. Hammick was requested to see a boy in Plymouth, of a scrofulous aspect, in whom there was no union six weeks after a fracture of the leg; the limb had scarcely swelled, and there had been but very little pain. Mr. H. ordered the leg to be moved every day without any splints, and after their re-adjustment, the lad was to quit his bed for the sofa, and attempt to walk about on crutches, carrying the fractured leg well secured in a sling. Considerable inflammation of the limb was the consequence of this treatment; every thing went on well, and the bone soon became perfectly united.

Union may be prevented by the system being under the influence of the secondary symptoms of syphilis, and in many cases the patients did well on eradicating that disease. A marine was in the Plymouth Hospital for seven months with a fracture of the leg, which would not unite. Our author discovered that he had been in the house, twelve months previously, for a bad venereal complaint, and that, after his dismissal, he had suffered under secondary symptoms. This "was quite sufficient to put him under a course of mercury," and before the soreness of his mouth went off, his leg was firmly united.

"Remember, however, that although mercury will effect a cure by uniting the bone, when the patient is labouring under the distress of the venereal virus in the system, or what are called secondary symptoms, yet it will sometimes, when used for the treatment of the primary ones, retard it, by the debility it produces; yet mercury will frequently be required by patients who never had any syphilitic taint, not only to act as an alterative, but even it will be necessary to push it to a considerable extent before union of a fractured bone will take place. Many of you recollect a marine sent in from head quarters, for a fracture of the leg which had occurred seventeen weeks previously, and where no union had taken place. He was apparently in perfect health, never had had any venereal taint, and was free from all appearance of disease. The camphorated mercurial ointment was locally applied till salivation was produced, when the callus fully and firmly formed, and the cure was effected. This, however, so commonly occurs, that it will not be necessary to trouble you with the detail of any more cases in confirmation of it." 118.

Scurvy, as might naturally be expected, will prevent union, nay, more than that, it will dissolve it after it has been formed for many years. In the siege of Gibraltar, when this disease made great ravages, bones became separated which had been a length of time united. And, what wonder that a part of the human fabric, which has seen but a few Winters, should give way under the ravages of a disease which breaks down the whole constitution of the frame, and unknits its firmest sinews! New growths can never boast of the vital power possessed by those portions which have grown with the body's growth and strengthened with its strength, for it is well known that tumours or formations, however free from malignancy they may be, have ever a tendency to run into ulceration, in other words to die, under the influence of comparatively slight causes of injury. But this en passant. Not sea-scurvy only, but that scorbutic or cachectic condition which bad diet, bad air, or a bad constitution will kindle up at home, has a mischievous effect on the process of union. Mr. Hammick knew a gentleman, of a very scrofulous habit, who had his leg broken by the kick of a horse. He fell into the greatest state of exhaustion, had hæmorrhagies from various parts, his teeth became loose, his limbs swelled, in short, he had all the symptoms of sea-scurvy; the bone never united; and after a long period of great suffering he died.

"You often see among the poor that bad diet and foul air, from confinement in ill ventilated dark rooms, or under-ground cellars, will prevent union, when by giving them more nourishment, or even removing them into purer air, they will rapidly recover their strength, and the limb will become firm. In the beginning of my service in this hospital, when we were exceedingly crowded with patients, it was the custom to select the extreme cases of surgery for the same wards, and not to distribute them about in different ones, as I have done for some years past; and at that period it was no unusual thing to find that union in fracture was very much retarded, and on an average took two or three weeks more than at present." 120.

Pregnancy, it is well known, will frequently prevent the union of a broken bone for a time, just as it will put a temporary stop to the morbid process of disease, for instance, that of phthisis. Mr. Hammick has seen three cases of this kind, in all of which union was ultimately effected. We saw a very remarkable instance of the same thing about two years ago, but in spite of every measure that could be devised, no union was obtained, and the patient remains afflicted with an artificial joint in the humerus.

The causes, then, of non-union being various, and we believe there are more than those alluded to by Mr. Hammick, some discretion must be employed in the treatment.* Supposing, however, that every thing short of an operation of some kind, has failed, and that one must be selected :—We have the choice of four—one to cut down and irritate; another to cut down and apply the actual cautery; a third to pass a seton between the ends of the bone; and a fourth to cut down and remove those ends.

"The first, of cutting down and irritating the ends of the bone, cannot be of any advantage: when an artificial joint is formed, the ends of the fractured bone are covered with cartilage, sometimes united by a ligamento-cartilaginous substance, having occasionally a perfect capsule, within which is a fluid very similar to the true synovia. Now, cutting into this, and irritating the ends of the bone, can never be of any service,—it is impossible for them to unite whilst the cartilage remains. But you will be told that this operation has sometimes succeeded. Yes, it certainly has; but, in my opinion, when it has, it has been performed without being required; that a regular artificial joint, such as above described, had not existed; that the ends of the bone merely lay without union, which, by discovering the cause, and adopting a suitable treatment, might have firmly united them." 123.

We agree with our author, that if the case really requires our cutting down upon the ends of the bone, it also requires something more than our merely "irritating them." This is sheer child's play; it is giving the patient all the pain, and much of the danger, of a severe operation, without its proportionate advantages.

In illustration of the second operation, that of applying the actual cautery to the ends of the bone, a case is related by Mr. Hammick. A lady with ununited fracture came from Ireland to London, and was received into one of the hospitals of the metropolis. At the suggestion of "a French surgeon," an incision was made on the outside of the arm, down to the fractured ends of bone, and the actual cautery thrust in between them. The pain thus

* The observations on non-union by Mr. Amesbury, and the cases adduced by that gentleman, have proved that none of the following operations are required nearly so often as they were before the introduction of his various apparatus. We have made some observations on this subject in a former Number of the Journal, and need not go over the ground again. By the bye, Mr. Hammick takes no notice of a method which is very often of service in promoting union, we mean the application of blisters.—Ed.

produced was indescribable, and such was the inflammation and sloughing which succeeded, that the patient's life was in considerable danger for many days. She survived, however, but her condition is worse now than before the operation, for the fingers have become so drawn up and weakened, and at times so painful, that her limb is worse than useless.

Mr. Hammick does not see how the seton can do any good, nor can he say what he would wound if he employed it. In his last case he would have "exactly passed it through the ossified artery between the ends of the bone," and a most unlucky business he would have made of it. Although our author cannot see how the seton can do good, it is quite certain that it has done so, and that not unfrequently. Theories and prejudices *should* give way to facts, but we remember the old adage, that "none so blind as those who will not see."

"The last method of cutting down and removing the ends of the bone, is certainly the one most likely to succeed, but then it is a very severe undertaking. You must recollect that the failure of union has been in a case of simple fracture; that, by this operation, you convert it into a bad compound one; for what do you do? You cut down to the ends of the broken bone, turn them out through the wound, and saw them off; to do which the wound must be pretty extensive, otherwise your saw would lacerate and jag the integuments and muscles, particularly if it be of the thigh. Now, when you have a compound fracture, with protrusion of the bone, the end of which it is desirable to remove, you very well know how difficult it is often to save the limb, and at the very best what a formidable case of surgery it is: then how much more so must that case be where you have to turn out both ends of the bone, and saw them off? Some surgeons have only removed one end, but they had better have done nothing at all; because whilst one remains covered with cartilage, union can no more take place than if they were both to remain so. It is certain that bone cannot unite to cartilage: the ossific anchylosis of the knee-joint, for instance, can never take place until the cartilage covering the two bones to be united, as the femur and tibia, be so far destroyed as to allow of bone uniting to bone; and, consequently, the same process must take place where union has failed after fracture, from their ends being tipped with cartilage. After all, however, this operation of cutting down and sawing off the ends of the fractured bones, is the only one that can, I think, succeed, supposing that a perfect artificial joint is established. I have never performed it; and very strong and weighty reasons must arise to bring my mind to do it. Had we attempted it in our last patient, as soon as I had introduced my scalpel between the ends of the bone, to divide the ligamento-cartilaginous connexion, I should have cut through, without any possibility of avoiding it, that large ossified artery running between the broken portions of the thigh; and you saw with what difficulty I secured the vessel even when the limb was off, and nothing but a lucky passage of the needle did it; how much more, then, would the danger have been increased with the limb on. The chances are that amputation must have been immediately performed, under very discouraging circumstances; or, if we could have stopped the blood without it, we should have considered it fortunate." 127.

Our author has thus disposed of all the operations for an artificial joint after ununited fracture, and as one is inefficient, another too severe, a third of no use, and a fourth pregnant with danger, it follows that he would employ none or neither. Let us see the substitute found by Mr. Hammick:

"From this detail you observe that we have grounds to justify us in saying, that there are many more causes for a failure of union in fractured bones than are commonly imagined; that by finding out the cause you may almost invariably remedy it, and obtain a cure; that where operations have been successful, a longer delay and other means would have probably rendered them unnecessary; and that when all other judicious and well-selected modes fail, it is more than doubtful whether any operation short of amputation ought to be employed. In this case of Sheriden's, which required amputation, you have seen the ossified state of the arteries of the whole of the lower extremity; and it is highly satisfactory for us to know, that nothing whatever could have produced a union of the bone, and that the taking off the limb was the only chance the patient had." 127.

Thus then amputation is the salve which our author would apply, rejecting all the other nostrums to which we have adverted. Now really we do think that any one of the operations formerly described is preferable to this dernier resort, this opprobrium of surgery, and we suspect that patients would be commonly of our opinion; such a cure for ununited fracture is like a wooden leg for the removal of corns, effectual, but unpleasant. We cannot attach much importance to the thrice-cited argument of the ossified artery, inasmuch as in all patients blood-vessels are not in that condition, and to frame general rules on the exception appears to us to be but indifferent reasoning.

Our limits now compel us to desist, though many more practical remarks might be culled from Mr. Hammick's book. We are not in the habit of winding up our analyses with those little bits of oracular judgment, like cheese after meat, with which some of our contemporaries are apt to favour the author and their readers. Still we may say of Mr. Hammick's work, that it contains too many practical hints to deserve to be overlooked by the surgeon engaged in practice, and that it is too discursive and imperfect ever to be adapted for a standard work to the surgical student. It is certainly not distinguished for classical lore, peculiar elegance of language, nor much acquaintance with the practice of contemporaries; indeed we believe there is scarcely a reference to a brother surgeon from the first page to the last. This might be explained were it worth while, but it is not; and we therefore conclude by recommending the perusal of the work to such of our surgical brethren as have a little time upon their hands, and are already tolerably acquainted with surgery as it is.

